# Glucocorticoid guides mobilization of bone marrow stem/progenitor cells via FPR and CXCR4 coupling

**DOI:** 10.1186/s13287-020-02071-1

**Published:** 2021-01-07

**Authors:** Wenting Gao, Xuetao Yang, Juan Du, Haiyan Wang, Hejiang Zhong, Jianxin Jiang, Ce Yang

**Affiliations:** 1 State Key Laboratory of Trauma, Burns and Combined Injury, Research Institute of Surgery, Daping Hospital, Third Military Medical University, Chongqing, 400042 People’s Republic of China; 2 Department of Cardiovascular Surgery, First Affiliated Hospital of Baotou Medical College, Baotou, 014000 Inner Mongolia People’s Republic of China; 3 Chinese PLA 952th Hospital, Geermu, 816000 Qinghai People’s Republic of China; 4 Department of Anesthesiology, Xinqiao Hospital, Third Military Medical University, Chongqing, 400037 People’s Republic of China

**Keywords:** Bone marrow, Corticotropin-releasing hormone, Glucocorticoids, Hypothalamic-pituitary-adrenal (HPA) axis, Formyl peptide receptor, Stem/progenitor cells, Chemotaxis

## Abstract

**Background:**

Our previous studies have proved the efficient exogenous repairing responses via bone marrow stem and progenitor cells (BMSPCs). However, the trafficking of endogenous bone marrow stem and progenitor cells to and from the bone marrow (BM) is a highly regulated process that remains to be elucidated. We aimed to study the relative importance of the hypothalamic-pituitary-adrenal (HPA) axis in the glucocorticoid-induced BMSPC mobilization.

**Methods:**

The circulating mesenchymal stem cells (MSCs) and endothelial progenitor cells (EPCs) were examined in Crh (+/+, −/−) mice after running stress or glucocorticoid mini-infusion. The MSCs and EPCs were investigated ex vivo after treatment with glucocorticoid and glucocorticoid receptor (GR) antagonist, RU486. The expression of chemotaxis receptors, N-formyl peptide receptor (FPR), and Cys-X-Cys receptor 4 (CXCR4) of MSCs and EPCs as well as their colocalization were investigated after treatment with glucocorticoid, glucocorticoid receptor (GR) antagonist (RU486), and FPR antagonist (Cyclosporin H).

**Results:**

Forced running stress increased circulating MSCs and EPCs in mice, which was blunted when Crh was knocked out, and positively related to the levels of serum glucocorticoid. Prolonged glucocorticoid mini-infusion imitated the stress-induced increase in circulating MSCs and EPCs in Crh^+/+^ mice and rescued the impaired mobilization in circulating MSCs and EPCs in Crh^−/−^ mice. Meanwhile, glucocorticoid promoted the chemotaxis of MSCs and EPCs ex vivo via GR, inhibited by RU486 (10 μM). Concurrently, glucocorticoid increased the expression of FPR of MSCs and EPCs, but inhibited their expression of CXCR4, followed by their changing colocalization in the cytoplasm. The GC-induced colocalization of FPR and CXCR4 was blunted by Cyclosporin H (1 μM).

**Conclusion:**

Glucocorticoid-induced CXCR4-FPR responsiveness selectively guides the mobilization of BMSPCs, which is essential to functional tissue repair.

**Graphical abstract:**

Schematic view of the role of glucocorticoid on the mobilization of bone marrow-derived stem/progenitor cells subsets in the present study. The HPA axis activation promotes the release of glucocorticoid, which regulates the directional migration of MSCs and EPCs mainly via GR. The possible mechanisms refer to the signal coupling of FPR and CXCR4. Their two-sided changes regulated by glucocorticoid are involved in the egress of MSCs and EPCs from BM, which is helpful for wound healing. MSCs, mesenchymal stem cells; EPCs, endothelial progenitor cells. 
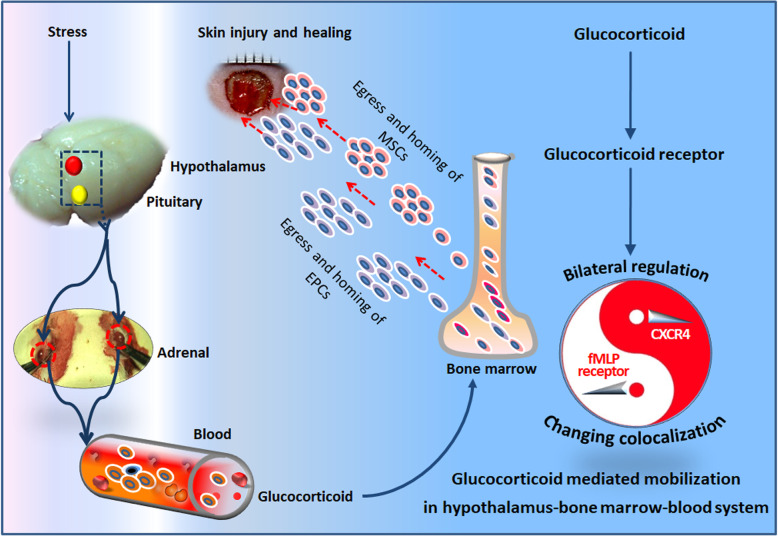

**Supplementary Information:**

The online version contains supplementary material available at 10.1186/s13287-020-02071-1.

## Introduction

In adult organisms, stem and progenitor cells localized primarily in the bone marrow (BM) have the ability to mobilize and differentiate into multiple cell phenotypes in injury. Among these subsets, bone marrow mesenchymal stem cells (BMSCs) and endothelial progenitor cells (EPCs) have the capacity to promote the repair of injured tissues [1–3]. Recently, regarding a serious practical and technical bottleneck associated with harvesting, isolation, in vitro expansion, identification, and engraftment of exogenous stem cells in clinical trials, mobilization of efficient amount of BM stem and progenitor cells (BMSPCs) is proposed to overcome this insufficiency in the repair of injured tissues and organs. To this end, the mobilization of BMSPCs mainly depends on the mobilizing agents, i.e., growth factors, C-X-C motif (CXC) chemokines, CXC receptor 4 (CXCR4) antagonist, granulocyte colony-stimulating factor (G-CSF), and dimethyloxallyl glycine (DMOG) [4–8]. However, they may result in ostalgia, headache, acratia, and even acute lung injury among 30% of patients [9]. More importantly, nearly 40% of patients, especially those who received anticancer therapies, are insensitive to these mobilizers.

Recent studies have shown that neuroendocrine responses play an important role in the regulation of BM-derived cells in stress conditions (trauma, burn, and hemorrhagic shock) [10–12]. The hypothalamus-pituitary-adrenal (HPA) and sympathetico-adrenomedullary (SAM) axes, coordinated by corticotropin-releasing hormone (CRH) [13, 14], might be intrinsically related to the outflow of BM-derived cells [15–17] and repairing activities after injury [18]. The number of circulating BM stem cell subsets is increased in trauma patients [19]. The pharmacological concentrations of glucocorticoid analog, hydrocortisone, could also mobilize the BM colony-forming cells into the peripheral blood [20]. Furthermore, in vitro studies have further revealed that dexamethasone could enhance the migration and chemotaxis of human mesenchymal stem cells and promote electrical impulse-simulated wound healing [21]. Hence, the activation of the HPA axis might have an intrinsic relation with the mobilization of BMSPCs.

Concerning the possible mechanisms for the mobilization of BMSPCs, previous studies focus on the stromal cell-derived factor 1α (SDF-1α) and CXCR4 (Cys-X-Cys receptor 4) [22–24], which help the store of stem cells in the BM niche. The disrupted interactions between SDF-1α and CXCR4 promote their initial release followed by active migration across the BM sinusoidal endothelium. CXCR4 was upregulated by G-CSF [22]. But during the activation of the HPA axis, the levels of G-CSF did not elevate [25], not supporting the chemotaxis of CXCR4 expressing BMSPCs toward injured areas with high SDF-1α concentration [10]. Additionally, researchers further found that N-formyl peptide receptor (FPR), a G protein-coupled receptor expressed in BM-derived cells [26, 27], played a key role in innate immunity, inflammation, and repair. Its ligand, N-formyl peptide (FP), is released through mitochondria disintegration or bacterial protein degradation in trauma and infections. Glucocorticoid could enhance the FPR expression and promote the chemotaxis in monocytes [28]. In addition, Annexin-1, regulated by glucocorticoid, was also an agonist of FPR. FPR and CXCR4 might exhibit the G protein-mediated interrelated scope. Previous studies showed that FPR could be involved in the modulation of CXCR4 by heterologous desensitization [29, 30].

Therefore, we hypothesized that HPA activation contributes to the mobilization of BM stem cells by modulating the chemotaxis response via FPR and CXCR4, favoring the migration toward injured tissues. Thus, we conduct studies in genetically modified Crh^−/−^ mice, which were succumbed to acute stress to induce HPA activation: (1) to confirm responses of the BMSPC mobilization between Crh^+/+^ and Crh^−/−^ mice and the origin of these stem cells, (2) in vitro and in vivo to determine the effects of glucocorticoid on the N-formyl peptide-induced chemotaxis in BMSPCs and its relation to FPR and CXCR4, and (3) to examine the role of FPR and CXCR4 coupling in the processes of mobilization of BM stem cells.

## Materials and methods

### Ethical approval

This study was approved by the Animal Care and Use Committee of the Third Military Medical University (Chongqing, China) that follows the ethical principles and guidelines for scientific experiments on animals of the Swiss Academy of Medical Sciences. This includes minimizing the number of animals for the experiment and taking measures to minimize their suffering. The number of animals enrolled in the present study was decided by power analysis.

### Animal management

Healthy Crh^+/−^ mice (Jackson Labs, Bar Harbor, ME, USA) (6 to 8 weeks and 22 to 30 g) were housed and cared for in the Experimental Animal Center of Daping Hospital of the Third Military Medical University, Chongqing, China. Crh^+/+^ and Crh^−/−^ mice used in our experiments have derived from Crh^+/−^ × Crh^+/−^ matings [31]. Genotyping in the offspring was performed using DNA obtained from post-weaning tail biopsies. PCR products were sequenced to confirm the presence or absence of murine Crh DNA in offspring (Crh^+/+^, Crh^−/−^). The animals were kept in a pathogen-free room under the controlled temperature (22–26 °C), humidity (45–55%), and lighting (12-h light-dark cycles) with free access to standard laboratory food and water.

### Model preparation

Mice were forced to run on a 6-lane treadmill (Model No. FT-200, Taimeng Instruments, Inc., Chengdu, China) by a grid at the back of the treadmill lane delivering a mild electrical shock (1 mA). Crh^+/+^ and Crh^−/−^ mice were subjected to running stress in accordance with a modified procedure [32]. Briefly, after 1 day of adaptation to the laboratory environment, the mice were divided into two groups respectively: (1) controls (Con), mice acclimatized to treadmill exercise but not run on the experiment day, and (2) stress group (St), mice were acclimatized to treadmill exercise and run on the experiment day. Con and St mice were exercised during the light cycle (10 min at 5–15 m/min; 0° slope) on an animal treadmill for 2 consecutive days. The acclimatization process was used to acquaint the animals with the treadmill and to ensure that the St mice would run in the experiment days. Then, all the mice were kept in the cages for 1 day to recess. On the experiment day, St mice were exercised on the animal treadmill for 2 h at 15 m/min, 6° slope [33], and Con mice were kept in the cages exposing to the noise and the trembling of the animal treadmill simultaneously. All animal experiments were finished at 08:30–11:30 during the day to avoid the interference of circadian rhythm on the BM microenvironment.

### Blood sample collection

One hour after the exercise session, the mice were immediately anesthetized with ethylether, and removed the eyeballs from the socket. Blood collection was performed from the orbital sinus. This protocol was followed to account for any discrepancy in hormone levels among the groups with respect to the stress treatment. Blood was collected into 1.5-ml heparinized plastic conical tubes and placed immediately on ice to maintain the viability and phenotype of cells. Blood samples were centrifuged at 700*g* for 10 min at 4 °C, the plasma from each mouse was placed in a cryovial and stored at − 70 °C for corticosterone analyses, and blood cells were prepared for flow cytometric analyses (FACS).

### Prolonged mini-infusion

The major glucocorticoid in mice is corticosterone, which was used to investigate the role of glucocorticoid supplement for the Crh^+/+^ and Crh^−/−^ mice. Corticosterone (Sigma, St. Louis, MO, USA) was dissolved in absolute alcohol at a concentration of 20 mg/ml and stored at − 20 °C until use. The stock solution was diluted to working solution using 0.9% sodium chloride solution and intraperitoneally infused to Crh^+/+^ or Crh^−/−^ mice continuously with 10 mg/kg body weight in 2 h using a micropump (Model No. LSP10-1B, Lange Instruments, Inc., China). Control animals were infused intraperitoneally with an equal volume of solvent without corticosterone simultaneously.

### Stimulation of the whole blood

To investigate the effect of corticosterone on the number of MSCs and EPCs in the peripheral blood, 1.5 ml of whole blood taken out from the Crh^+/+^ mice was immediately stimulated with 75 ng/ml corticosterone in a sample mixer at 37 °C for 3 h.

### Flow cytometric analyses

The isolated blood cells were suspended in staining buffer (2% heat-inactivated fetal calf serum, 0.09% sodium azide in Dulbecco’s PBS; Invitrogen) and blocked using purified rat anti-mice Fc block (BD Pharmingen, San Jose, CA). The viable cell population was analyzed for MSCs with Sca-1-PE(0.2 mg/ml, BioLegend) and CD49a-FITC (0.2 mg/ml, BioLegend) antibody and for EPCs with CD133-PE (0.2 mg/ml, eBioscience) and VEGFR-2-FITC (0.2 mg/ml, Flk-1; BD Parmingen) antibody as previously described respectively [34–37]. Isotype-identical antibodies served as controls to determine background staining in every experiment. Flow cytometry was performed using the Beckman Coulter Cytomics FC500 (BD Biosciences, USA).

### Assay of plasma corticosterone

The plasma samples were collected and disposed as described above at 1 h after running stress. The corticosterone levels in the plasma were determined with enzyme-linked immunosorbent assay (ELISA) according to the manufacturer’s instructions (R&D Systems, Minneapolis, MN, USA). Each experiment was conducted in duplicate. The concentration of corticosterone was detected at 450 nm using a microplate reader (Tecan, Infinite 2000, Austria). The sensitivity of this immunoassay was 4 pg/ml.

### Isolation, purification, and identification of MSCs and EPCs

Mice BM MSCs were collected and purified by a modified method [38]. The complete medium was changed every 3 days. Cells were detached by 0.125% trypsin-0.01% EDTA and under passage into fresh culture flasks at a ratio of 1:3 upon reaching confluence. Cells were incubated at 37 °C in a humidified incubator with 5% CO_2_. Mouse BM EPCs were isolated and cultured as described previously [39]. After 7 days in culture, EPCs were identified by uptake of 1,1′-dioctadecyl-3,3,3′, 3′-tetramethylindocarbocyanine-labeled acetylated LDL (DiLDL, 2.4 μg/ml; Cell Systems) and staining with fluorescein isothiocyanate-labeled lectin (10 μg/ml; Sigma) and used for other experiments.

### Chemotaxis assay of MSCs and EPCs

Chemotaxis of cultured EPCs and MSCs was assessed using a standard 48-well chemotaxis chamber (Neuro Probe, Cabin John, MD) with 5- or 8-μm pores with a modified technique described previously [40]. Briefly, cell suspensions (5, 0000) of EPCs and MSCs in BM were added to the upper chemotaxis chambers with corticosterone ranging from 0 to 1500 ng/ml overlying wells containing binding medium with N-formyl methionyl leucyl phenylalanine (fMLP) (0 ng/ml, 100 ng/ml). Following incubation (4 h) at 37 °C, 5% CO_2_. The chemotactic index, which represents the total number of cells counted at random from 16 fields on the lower face of the filters, was calculated under microscopy (× 200).

### Treatment of MSCs and EPCs with glucocorticoid, mifepristone, and Cyclosporin H

MSCs or EPCs (1 × 10^6^ cells/well) incubated in 6-well Costar plates overnight were washed with 0.01 M PBS, and corticosterone ranging from 0 to 1500 ng/ml was added to the respective wells, and cells were incubated for 4 h at 37 °C in a humidified incubator with 5% CO_2_. The reactions were terminated by aspirating the medium. Mifepristone (Sigma, St. Louis, MO, USA), a GR antagonist, was dissolved in 95% ethanol at a final concentration of 10 μM at 37 °C for 60 min before corticosterone was added. MSCs and EPCs were also incubated with 1 μM Cyclosporin H (Santa Cruz Biotechnology, Inc.), a potent and selective FPR antagonist, for 30 min before corticosterone was added.

### Quantitative real-time PCR

Total RNA in MSCs and EPCs was isolated with TRIzol Reagent according to the standard procedures (Invitrogen, Karlsruhe, Germany). Potentially contaminated DNA was removed by DNAase I treatment followed by a purification step with the RNAeasy Mini Kit (Qiagen, Hilden, Germany). One microgram of total RNA was transcribed into cDNA with Superscript II Reverse Transcriptase (Invitrogen, Carlsbad, CA, USA) by Oilgo-dT priming. The cDNA products were used immediately for SYBR green (Applied Biosystems, Darmstadt, Germany) real-time RT-PCR for FPR and CXCR4. cDNA was amplified using gene-specific primers (Sangon Technology Inc., Shanghai, China) described as follows: FPR (sense: 5′-CTGCTGGCTACATCGTTCT-3′, antisense: 5′-TGCACATGAACCAACCAAAT-3′), CXCR4 (sense: 5′-AACCACCACGGCTGTAGA-3′, antisense: 5′-CTCCTTAGCTTCTTCTGGTA-3′), and β-actin (sense: 5′-GCCCAGAGCAAGAGAGGTA-3′, antisense: 5′-CCTCGTAGATGGGCACAGT-3′). Relative quantification was performed using the ΔCt method which results in ratios between target genes and a housekeeping reference gene, β-actin. The relative expression was calculated as 2^−△△Ct^, [△△Ct = (Ct_target gene_ − Ct_β-actin_) Exp − (Ct_target gene_ − Ct_β-actin_) Con].

### Immunofluorescence

The cells were fixed in 4% paraformaldehyde in 0.01 M PBS (pH 7.4) for 10 min. Immunocytochemical studies were performed as described previously [31]. Briefly, incubate cells in the 1:200 diluted FPR rabbit polyclonal antibody (Santa Cruz Biotechnology, Inc.), CXCR4 rabbit polyclonal antibody (Santa Cruz Biotechnology, Inc.), or goat polyclonal antibody (Boster biotechnology, Inc.) in 1% BSA and 10% goat or goat plus donkey serum in PBS in a humidified chamber for 1 h at 37 °C and then overnight at 4 °C. Cells in the negative control group were incubated with the corresponding solution without antibody in the same condition. Decant the solution and wash the cells with PBS three times for 5 min. Incubate cells with the 1:400 diluted donkey anti-rabbit IgG, FITC 488 (lot number: LJ151862, Thermo), or donkey anti-goat IgG, AF594 (lot number: LJ151862, Thermo) in PBS for 1 h at 37 °C in dark. The mean fluorescence intensity representing the expression of FPR was calculated by the Leica confocal software (Leica, Germany).

### Colocalization analyses of FPR and CXCR4

The cells with double immunofluorescence staining of FPR and CXCR4 were further analyzed on the visible cell workstation (DeltaVision, USA). The full-thickness scanning of these immunofluorescent cells was carried out along the *Z*-axis of cells. Then, the results were further given deconvolution treatment to improve the quality of cell images. Finally, the three-dimensional reconstruction of these cells was acquired with the Immaris software. The colocalization of FPR and CXCR4 was assessed using Pearson’s coefficient in colocalized volume.

### Model of wound healing

Mice were anesthetized using pentobarbital sodium (40 mg/kg) and then shaved with an electric shaver so as to avoid injury. Shaved mice were cleaned with both depilatory and sterile saline before creating 5-mm diameter, full-thickness, circular wounds in the middle dorsal skin of each mouse. Immediately after injury, the mice were then kept in a sterile cage before the mice received daily exercise training as previously described [33]. Nonexercised but anesthetized, shaved, and wounded mice were also utilized for comparison. The wound area was measured directly from digital photographs using the Photoshop 7.0 software. Skin wound healing was assessed using the healing rate of the incisional wound, which was derived by the following formula: [1 − (current wound size/initial wound size)] × 100.

### Statistical analyses

The data are presented as mean ± SD. Paired and unpaired Student *t* tests and ANOVA for multiple comparisons were used where applicable. Post hoc comparisons were performed with the Neuman-Keuls test. Values of *P* < 0.05 were considered significant. All statistical calculations were performed using the SPSS18.0 software for Windows.

## Results

### Forced running stress increased circulating MSCs and EPCs in mice, which was blunted when Crh was knocked out

**Fig. 1:**
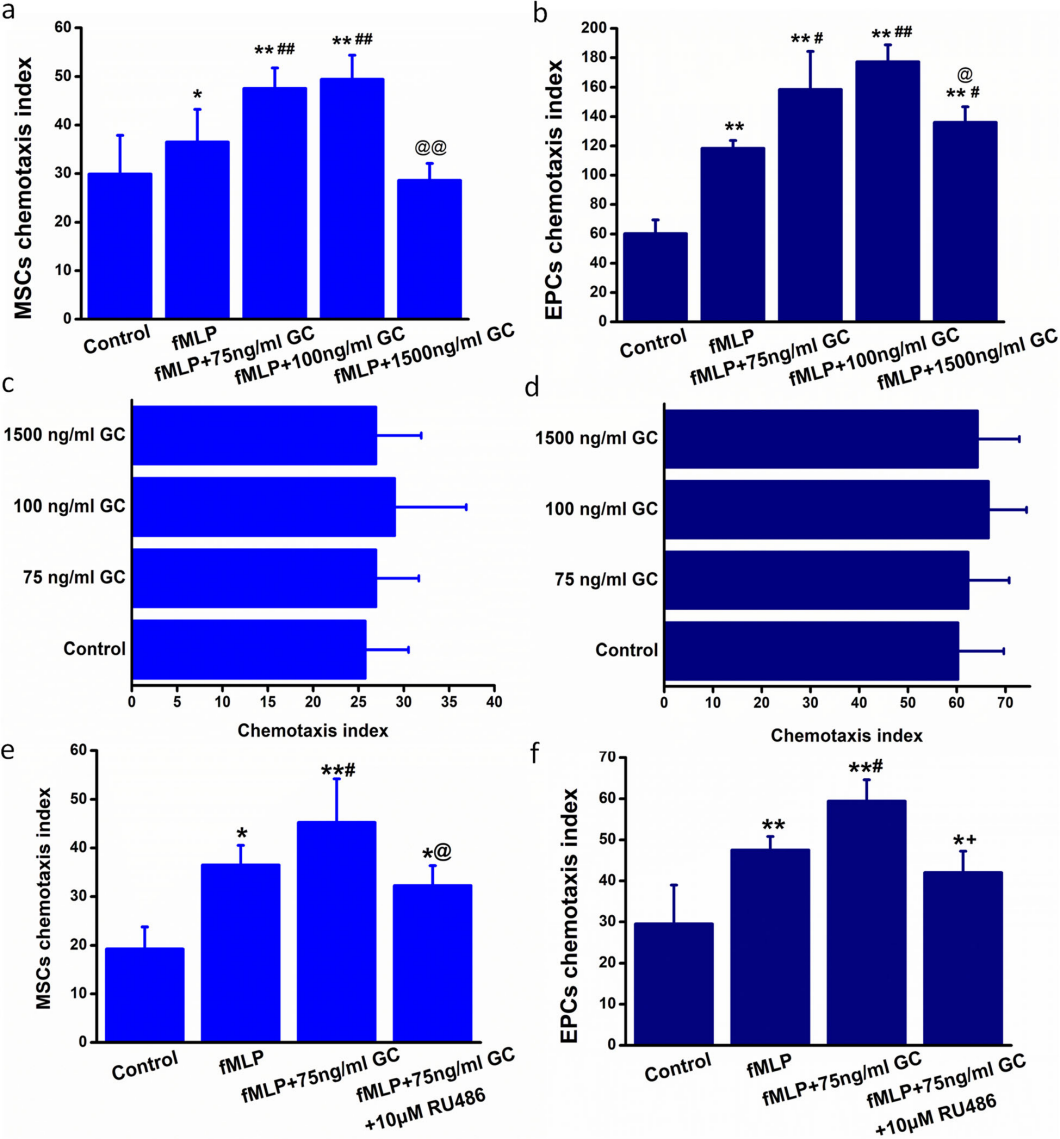
Glucocorticoid treatment promoted the chemotaxis of bone marrow-derived stem/progenitor cell subsets via glucocorticoid receptor. **a** GC promoted MSC chemotaxis toward fMLP. *P < 0.05, **P < 0.01 vs. the control group, ^# #^*P* < 0.01 vs. the fMLP group, ^@@^*P* < 0.01 vs. the fMLP + 75 ng/ml GC group and the fMLP + 100 ng/ml GC group. **b** GC promoted EPC chemotaxis toward fMLP. **P < 0.01 vs. the control group, ^#^*P* < 0.05, ^# #^*P* < 0.01 vs. the fMLP group, ^@^*P* < 0.05 vs. the fMLP + 100 ng/ml GC group. **c** GC could not promote MSC directional migration through transwell membrane within 3 h. **d** GC could not promote EPC directional migration through transwell membrane within 3 h. **e** RU486 inhibited GC-induced MSC chemotaxis toward fMLP. *P < 0.05, **P < 0.01 vs. the control group; ^#^*P* < 0.05 vs. the fMLP group, ^@^*P* < 0.05 vs. the fMLP + 75 ng/ml GC group. **f** RU486 inhibited GC-induced EPC chemotaxis toward fMLP. *P < 0.05, **P < 0.01 vs. the control group, ^#^*P* < 0.05 vs. the fMLP group, ^@^*P* < 0.05 vs. the fMLP + 75 ng/ml GC group. MSCs, mesenchymal stem cells; EPCs, endothelial progenitor cells; GC, glucocorticoid; fMLP, *N*-formyl methionyl leucyl phenylalanine. Data are mean ± SD of six representative observations

**Fig. 2:**
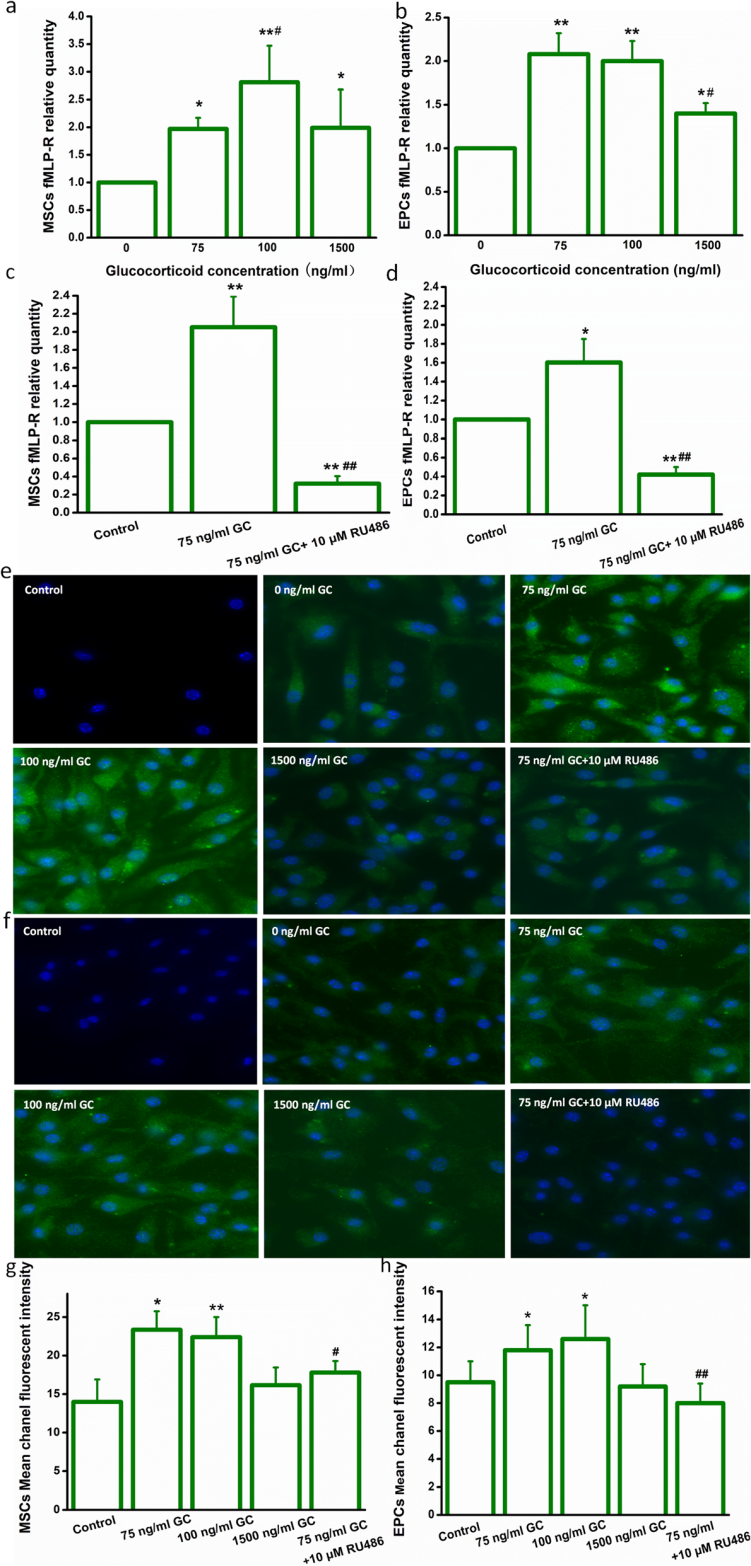
Glucocorticoid increased the FPR expression of bone marrow-derived stem/progenitor cell subsets via glucocorticoid receptor. **a** GC enhanced FPR mRNA expression in MSCs. *P < 0.05, **P < 0.01 vs. the control group, ^#^*P* < 0.05 vs. the 75 ng/ml group. **b** GC enhanced the FPR mRNA expression in EPCs. *P < 0.05, **P < 0.01 vs. the control group, ^#^*P* < 0.05 vs. the 75 ng/ml and 100 ng/ml groups. **c** RU486 inhibited GC-induced FPR mRNA expression in MSCs. **P < 0.01 vs. the control group, ^##^*P* < 0.01 vs. the 75 ng/ml group. **d** RU486 inhibited GC-induced FPR mRNA expression in EPCs. *P < 0.05, **P < 0.01 vs. the control group, ^##^*P* < 0.01 vs. the 75 ng/ml group. **e**, **g** FPR protein expression in MSCs. *P < 0.05, **P < 0.01 vs. the 0 ng/ml group. Control: the negative control. ^#^*P* < 0.05 vs. the 75 ng/ml group. **f**, **h** FPR protein expression in EPCs. *P < 0.05, **P < 0.01 vs. the 0 ng/ml group. Control: the negative control. ^#^*P* < 0.05, ^##^*P* < 0.01 vs. the 75 ng/ml group. MSCs, mesenchymal stem cells; EPCs, endothelial progenitor cells; GC, glucocorticoid; FPR, formyl peptide receptor. Data are mean ± SD of six representative observations

After forced running stress, plasma corticosterone in Crh^+/+^ mice increased significantly compared with controls (*P* < 0.01), but have no change in Crh^−/−^ mice (S Figure 1). Simultaneously, circulating MSCs and EPCs increased nearly 2-fold in Crh^+/+^ mice after stress but not in Crh^−/−^ mice, as shown in S Figure 2.

### Prolonged glucocorticoid mini-infusion imitated the stress-induced increase in circulating MSCs and EPCs in mice

**Fig. 3:**
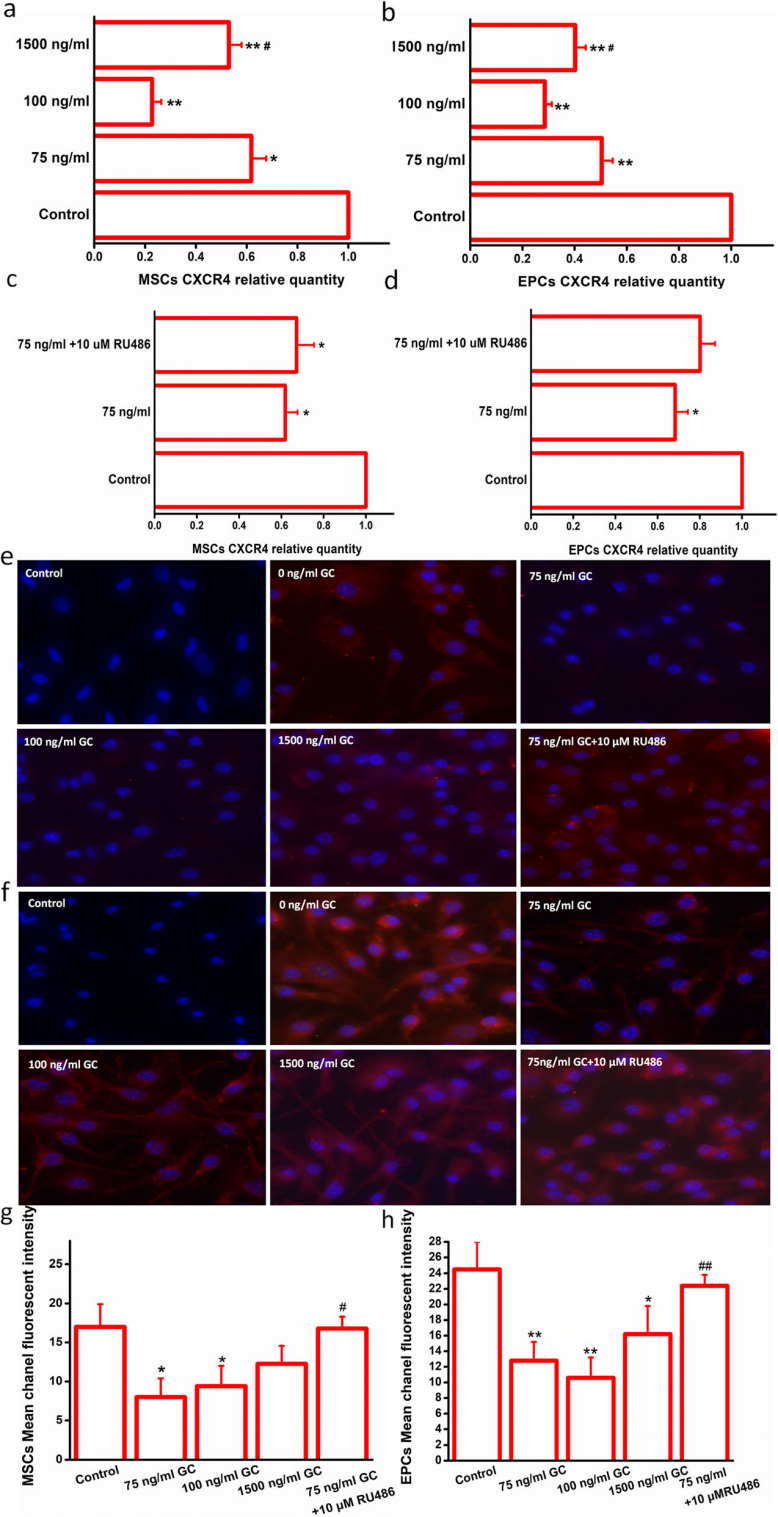
Glucocorticoid inhibited the CXCR4 expression of bone marrow-derived stem/progenitor cell subsets via FPR. **a** GC inhibited the CXCR4 mRNA expression in MSCs. *P < 0.05, **P < 0.01 vs. the control group. ^#^*P* < 0.05 vs. the 75 ng/ml group. **b** GC inhibited the CXCR4 mRNA expression in EPCs. **P < 0.01 vs. the control group. ^#^*P* < 0.05 vs. the 75 ng/ml and 100 ng/ml groups. **c** RU486 reversed the GC-induced CXCR4 mRNA expression in MSCs. *P < 0.05 vs. the control group. **d** RU486 reversed the GC-induced CXCR4 mRNA expression in EPCs. *P < 0.05 vs. the control group. **e**, **g** CXCR4 protein expression in MSCs. *P < 0.05 vs. the 0 ng/ml group. Control: the negative control. **f**, **h** CXCR4 protein expression in EPCs. *P < 0.05, **P < 0.01 vs. the 0 ng/ml group. Control: the negative control. ^##^*P* < 0.01 vs. the 75 ng/ml group. MSCs, mesenchymal stem cells; EPCs, endothelial progenitor cells; GC, glucocorticoid; FPR, formyl peptide receptor. Data are mean ± SD of six representative observations

**Fig. 4:**
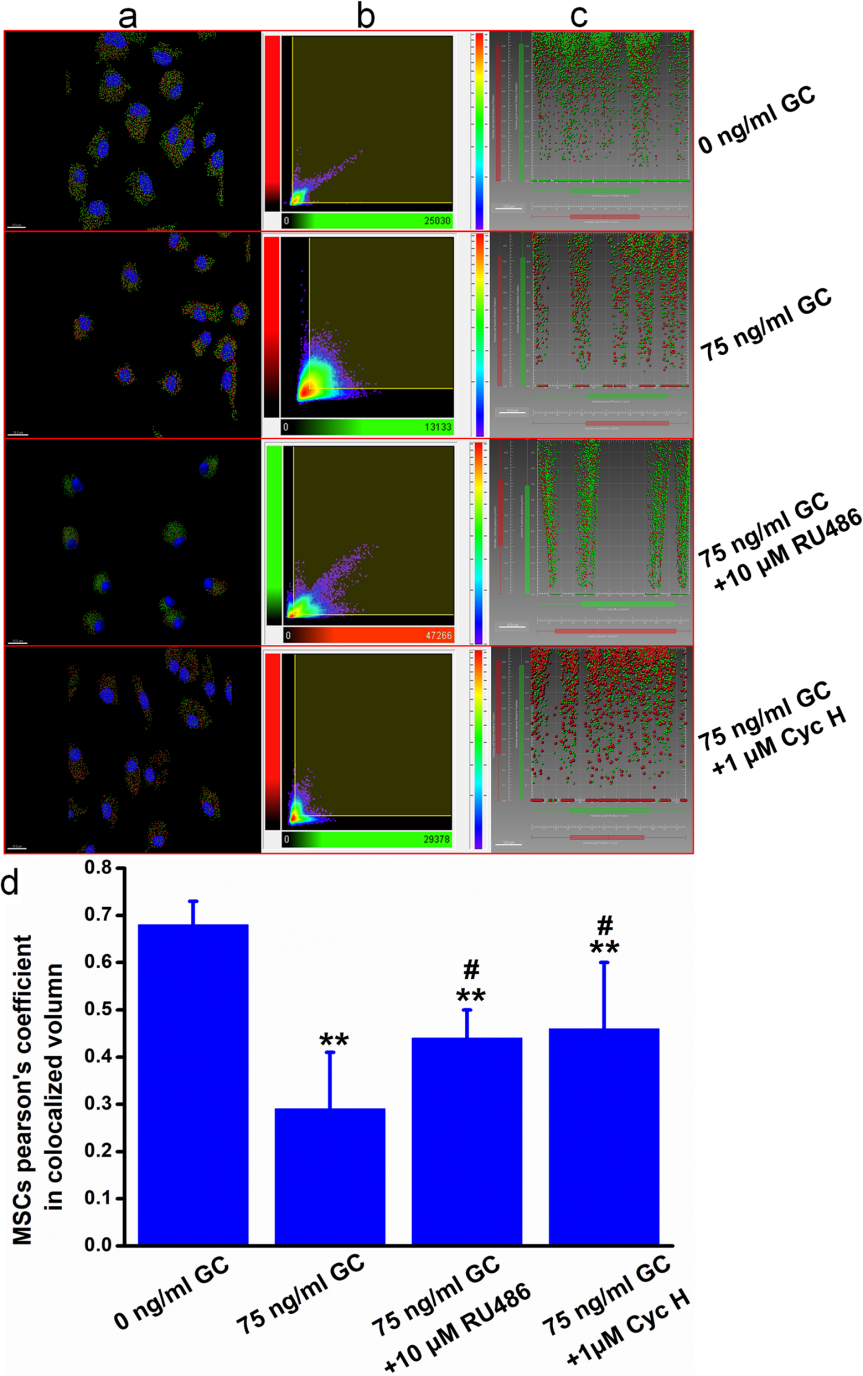
Glucocorticoid regulated the colocalization of FPR and CXCR4 of bone marrow-derived mesenchymal stem cells. **a** Colocalization of FPR and CXCR4 in MSCs after double immunofluorescence staining, deconvolution, and spotted treatment. **b** 2D histogram of Pearson’s coefficient in colocalized volume in MSCs. **c** Scatterplot of the vesicle relative position of FPR and CXCR4 after vantage treatment in MSCs. Pearson’s coefficient in colocalized volume was significantly decreased in MSCs after 75 ng/ml corticosterone treatment. When pretreated with RU486 (10 μm), Pearson’s coefficient in the colocalized volume of corticosterone-treated MSCs was obviously reversed compared to the 75 ng/ml GC group. Meanwhile, it was also reversed when pretreated with Cyc H (1 μm), a specific FPR antagonist. **P < 0.01 vs. the 0 ng/ml GC group, ^#^*P* < 0.05 vs. the 75 ng/ml group. Data are one of three representative observations

When the levels of plasma corticosterone were similar to those in mice 1 h after forced running stress, the number of circulating MSCs and EPCs showed a significant increase, which indicates that the elevated plasma corticosterone could facilitate the increase in circulating MSCs and EPCs directly (S Figure 3). Surprisingly, the blunted mobilization of circulating MSCs and EPCs was recovered significantly in Crh^−/−^ mice (S Figure 4), indicating the compensation effect of their mobilization from BM when enforced by glucocorticoid.

### The number of innate circulating MSCs and EPCs has no changes after ex vivo glucocorticoid stimulation within the experimental time

**Fig. 5:**
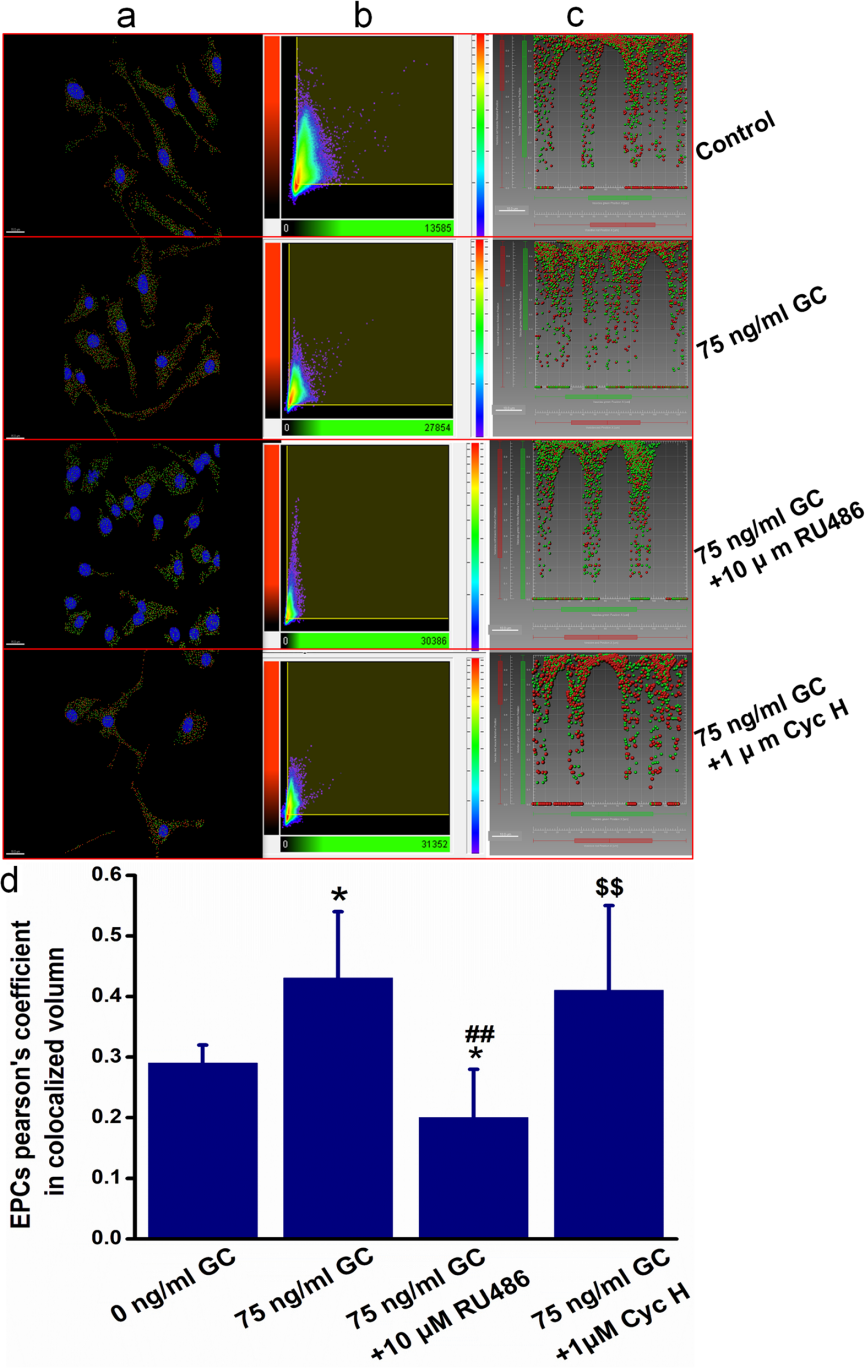
Glucocorticoid regulated the colocalization of FPR and CXCR4 of bone marrow-derived endothelial progenitor cells. **a** Colocalization of FPR and CXCR4 in EPCs after double immunofluorescence staining, deconvolution, and spotted treatment. **b** 2D histogram of Pearson’s coefficient in colocalized volume in EPCs. **c** Scatterplot of the vesicle relative position of FPR and CXCR4 after vantage treatment in EPCs. Pearson’s coefficient in colocalized volume was significantly increased in EPCs after 75 ng/ml corticosterone treatment (*P* < 0.05). When pretreated with RU486 (10 μm), Pearson’s coefficient in the colocalized volume of corticosterone-treated EPCs was obviously reversed compared to the GC group. Meanwhile, it was also reversed when pretreated with Cyc H (1 μm), a specific FPR antagonist, and more than that in the GC + RU486 group. *P < 0.05 vs. the 0 ng/ml GC group, ^##^*P* < 0.01 vs. the 75 ng/ml group, ^$$^*P* < 0.01 vs. the 75 ng/ml + 10 μM RU486 group. Data are one of three representative observations

To rule out the involvement of innate blood stem/progenitor cells, we further collected the whole blood in Crh^+/+^ mice and performed the ex vivo stimulation with similar stressful concentration of corticosterone (75 ng/ml) for 3 h, as previously mentioned in the forced running stress. However, there were no changes in the number of MSCs and EPCs (S Figure 5), indicating the mobilization of BM stem cells in the Crh^+/+^ mice is not related to their resident counterparts in the blood.

### Glucocorticoid promoted the mobilization of MSCs and EPCs via GR

**Fig. 6:**
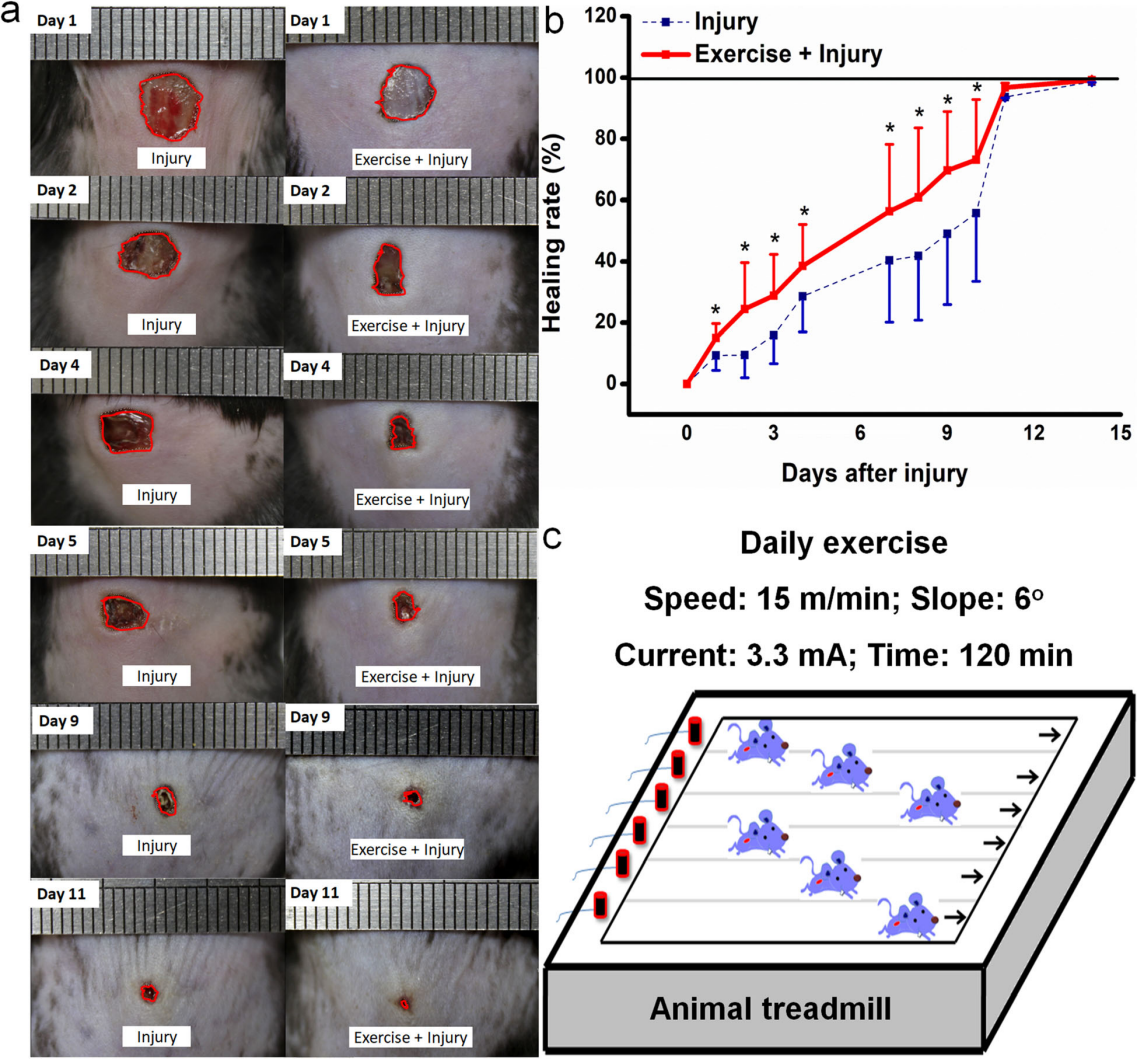
Moderate running exercise promoted wound healing in mice. **a** Photographs of representative wounds in mice at 1, 2, 4, 5, 9, and 11 days after injury. **b** Time-dependent increase in wound healing rate after daily appropriate exercises. *P < 0.05, **P < 0.01 vs. the control group. MSCs, mesenchymal stem cells; EPCs, endothelial progenitor cells. *n* = 8 per group. Data are mean ± SD

Consistent with the in vivo mobilization of BMSPCs in HPA axis activation, the stressful concentration of glucocorticoid could also enhance fMLP-mediated chemotaxis in MSCs and EPCs. As shown in Fig. 6, stressful concentrations of corticosterone could significantly augment the chemotaxis in both MSCs and EPCs toward fMLP. Furthermore, when pretreated with RU486 (10 μm), a specific GR antagonist, corticosterone-treated MSCs and EPCs showed a significant attenuation in fMLP-mediated chemotaxis (*P* < 0.05). Simultaneously, corticosterone could not promote the migration of MSCs and EPCs without fMLP (Fig. 1), indicating the increased chemotaxis might due to the enhanced interactions between fMLP and its corresponding receptor, FPR.

### Glucocorticoid increased the expression of FPR of MSCs and EPCs via GR

The expression of FPR was also increased when exposed to stressful concentrations of corticosterone for 3 h. The results shown in Fig. 2 reveal that FPR mRNA levels of MSCs and EPCs were significantly upregulated when treated with corticosterone, and more significantly, the FPR protein expression also increased, which are both inhibited by the RU486 pretreatment.

### Glucocorticoid inhibited the expression of CXCR4 of MSCs and EPCs via FPR

The mRNA and protein expression of CXCR4 in MSCs and EPCs were significantly inhibited after the corticosterone stimulation at the stressful concentrations, which are both reversed by the Cyclosporin H (1 μm) pretreatment (Fig. 3), suggesting the potential crosstalk between FPR and CXCR4 after acute stress.

### Glucocorticoid-induced colocalization of FPR and CXCR4 of MSCs and EPCs

Pearson’s coefficient in colocalized volume was significantly increased in MSCs while it was decreased in EPCs (*P* < 0.05, *P* < 0.01) after their double immunofluorescence staining, which is related to the changing amount and relative positions of these two receptors. Further, when pretreated with RU486 (10 μm), Pearson’s coefficient in the colocalized volume of corticosterone-treated MSCs and EPCs was obviously reversed compared to the GC group (*P* < 0.05). Meanwhile, it was also reversed when pretreated with Cyc H (1 um), a specific FPR antagonist, but more than that in the GC + RU486 group (*P* < 0.05) (Figs. 4 and 5).

### Moderate running exercise accelerated the wound healing in mice

Daily running exercise significantly improved the healing rate compared with nonexercised mice. The greatest effect of running exercise was observed at 9 days post-wounding (*P* < 0.05) (Fig. 6), indicating BMSPC mobilization initiated by HPA activation is helpful for wound healing and recovery.

## Discussion

Previous studies have proved the efficient exogenous repairing responses via BMSPCs [38, 41]. However, the trafficking of endogenous BMSPCs to and from BM is also a highly regulated process. Stem and progenitor cells can be forced out of the BM and might repair damaged tissues once mobilized into the blood. Our data revealed that HPA activation contributes to the mobilization of BMSPCs via glucocorticoid with the coupling of FPR and CXCR4 at least by modulating the chemotaxis response, favoring the migration toward injured tissues.

As the largest reservoir of stem/progenitor cells, BM possesses intrinsic relation with the neuroendocrine network. It might be an efficient measure for the tissue repair to initiate hormone-mediated endogenous repair courses via the mobilization of BMSPCs. In addition to the signals of the sympathetic nervous system and vagus for the mobilization [16, 42], our results firstly indicated that the HPA axis involved in the initiation of BMSPC mobilization other than those from local stem cell niches such as the liver, lungs, and spleen. Meanwhile, owing to the various types of progenitor cells within the peripheral blood, the mobilization by glucocorticoid is complicated. Since BMSCs are higher concentrated than mature rare cells, the role of rare cell types within the peripheral blood had not been investigated yet.

Our study indicated that the excitation of the HPA axis is related to the changes in circulating BMSPC number. To change the HPA responsiveness after stress, we used the Crh^−/−^ mice by the forced running stress. The elevation of circulating MSCs and EPCs was blunted, positively correlated to the alleviated levels of plasma glucocorticoid, indicating the releasing of adrenal glucocorticoid might directly modulate the BMSPC mobilization. Noteworthy, EPCs released from the BM are also mediated by eNOS-derived nitric oxide (NO) produced by the regulatory components of the BM microenvironment. Substances that increase NO bioavailability, like growth hormone (GH) and insulin growth factor-1 (IGF-1), increase EPC levels [43]. However, they were concurrently suppressed by elevated levels of glucocorticoids [44–46], indicating their limited role during these mobilizing processes after murine running stress.

To further confirm the glucocorticoid-mediated mobilization, mice were given a prolonged mini-infusion of corticosterone intraperitoneally to imitate the stressful state of the HPA axis. The increase in the number of circulating MSCs and EPCs was also found in Crh^+/+^ mice. Surprisingly, the impaired mobilizing ability due to blunted HPA axis responses was also supplemented in Crh^−/−^ mice after a similar treatment, suggesting the elevation of circulating MSCs and EPCs is mainly through HPA axis excitation and glucocorticoid releasing. In addition, to rule out the possibility that elevated corticosterone might change the numbers of innate circulating BMSCs and EPCs, the whole blood was collected and given stimulation of stressful concentration of corticosterone. Consequently, there are no changes in the number of circulating MSCs and EPCs, indicating that glucocorticoid might not induce the differentiation or dedifferentiation of resident circulating MSCs and EPCs, and the proliferation of circulating MSCs and EPCs are impossible during the experiment. Additionally, since the sensitivity to glucocorticoids differs not only among individuals but also within tissues of the same individual and even within the same cell during the cell cycle, the sensitivity to glucocorticoids on the mobilization of BMSCs should be considered. In view of the previous findings [47, 48], it may be related to the expression and affinity of glucocorticoid receptors, as well as glucocorticoid responsive elements in BMSCs. Also, the synergistic genomic and nongenomic effects of glucocorticoids in stress should be considered.

The ex vivo experiment further confirmed that the stressful concentrations of corticosterone promoted the chemotaxis of MSCs and EPCs while inhibited by RU486, an antagonist of the glucocorticoid receptor (GR). Also, the corticosterone did not by itself boost the directional migration of MSCs and EPCs without chemokines, but elicited egress from BM, indicating glucocorticoid mainly plays the enhancive effects during these courses with the aid of chemokines. These ex vivo and in vivo results demonstrate that the HPA axis excitation and glucocorticoid releasing were involved in the selective mobilization of BMSPCs through the GR pathway in a genomic dependant style.

Given the effects of glucocorticoid in BMSPC mobilization, the SDF-1α/CXCR4 axis has gained considerable recognition as a powerful mobilization power of BMSPCs [49]. The disruption of the SDF-1α/CXCR4 axis by G-CSF promoted the mobilization of hematopoietic progenitor cells (HPCs). But the plasma G-CSF did not increase in the HPA excitation during the exercises [25]. In combination with our results that the circuiting HPCs did not increase after forced running exercise (data not shown) but not treatment with physiological concentration of corticosterone [50], G-CSF-SDF-1α/CXCR4 is a classical pathway only for the mobilization of HPCs [51]. Concurrently, although the plasma levels of VEGF may elevate during the running stress, the acute administration of VEGF could not result in the mobilization of BMSPCs in response to the CXCR4 antagonist [51]. Therefore, the HPA activation selectively stimulated the mobilization of MSCs and EPCs in the BM through another pathway rather than SDF-1α/CXCR4 after running stress.

Meanwhile, MSCs and EPCs both expressed FPR, a typical chemotaxis receptor in immune defense against pathogens. FPR played an important role in host defense mechanisms due to its ability to interact with fMLP, produced in the tissue destruction, mitochondria disintegration, and bacterioprotein degradation [52]. FPR belongs to G protein-coupled receptors, similar to CXCR4. We found that the mRNA and protein expression of FPR were both upregulated in MSCs and EPCs, both inhibited by RU486, indicating FPR was a significant chemotaxis receptor in the BM mobilization. Concerning the colocalized manner of FPR and CXCR4 in the MSCs and EPCs, previous studies showed that FPR might modulate the CXCR4 expression via the receptor desensitization route [53], further indicating their intrinsic interactions after stress. Therefore, HPA excitation may promote the selective mobilization of BMSPCs via signaling coupling of FPR-CXCR4. Presently, if different doses of the reagents or different combinations of the reagents were injected, the dynamic changes of running stress hormones cannot be simulated objectively, and the in vivo pharmacokinetic properties and half-lives of different components will further increase the complexity of the analysis of experimental results. In addition to FPR and CXCR4, several other GPCRs expressed on the cell surface have been reported as potential biomarkers of osteoprogenitor cell subpopulation critical for both bone homeostasis and repair. Lgr6 and closely related Lgr4 are expressed in mouse bone marrow cells and bone marrow-derived mesenchymal stem cells [54]. Lgr5 may be a potential biomarker for the identification of a subpopulation of peripheral blood MSCs [48], which may function as sensors that would be activated in immune response or other stimuli. The origins of these newly identified cells in the blood and their therapeutic potential were also valuable to promote bone repair.

The present study has better demonstrated the elevated glucocorticoid facilitates the mobilization of BMSPCs via upregulating the expression of FPR, which in turn resulted in the complementary CXCR4 inhibition and low responsiveness to SDF-1. Through the signaling interactions of FPR and CXCR4, the BMSPCs were selectively mobilized in the circulation, directionally migrated toward the fMLP gradient in the injured tissues for repair. The improved wound healing in mouse skin was thought to be associated with the enhanced mobilization of BMSPCs [55] after appropriate stress (Graphical abstract). Hence, an appropriate stress response might guide the body’s stem/progenitor cells to exit their barracks (bone marrow and spleen), travel the circulation routes, and take a position in the potential battlefield (injured tissues and infected locus).

Wounding healing is a complicated course including tissue regeneration, proliferation of granulation tissue, and cicatrization. The promotion of wound healing is characterized by increase in healing velocity, reduction in healing time, and improvement of repairing quality. In our experiment, it is viewed that appropriate running exercise may at least increase the healing rate from day 3 to day 9. Clinically, it is valuable for the reduction in wound complication in the early phase of trauma, as well as a decrease in the medical cost. Additionally, besides the involvement of mobilization of MSCs and EPCs, increase in inflammatory cells (neutrophils, macrophages), proliferation of myofibroblasts and fibroblasts, and secretion of other neuroendocrine hormones (5-HT, angiotensin, and norepinephrine, etc.) may also be involved in these courses.

Some limitations need to be considered. We investigated the mobilization of BMSPCs via the flow cytometric analyses in vivo and chemotaxis assay ex vivo. Further numeric evidence of clonogenic activity per volume of blood seems a very solid evidence. However, the innate EPCs and MSCs in circulation may shield the number of judgment of BMSPCs after stress. The differentiation of MSCs and EPCs in clones is difficult in the present procedure. Secondly, the immunophenotype, colony-forming ability, proliferation and multi-differentiation capacity, and cytoskeletal actin of MSCs and EPCs should be evaluated. The changes of the BM microenvironment should also be paid close attention to as it is the main reservoir of adult stem/progenitor cells. These biological representations determine their fate in immunity and inflammation, as well as repair and regeneration. Thirdly, identification of potential target genes that CRH action through whole-genome gene expression profiling analysis comparing between wild-type and Crh knockout mice-derived MSCs and EPCs is in need, which may be instrumental to figuring out associated signaling pathways potentially regulating glucocorticoid-induced or stress-induced mobilization of MSCs and EPCs for more comprehensive analysis. Finally, the role of innate EPCs and MSCs in wound healing should be evaluated during the mobilization of BMSPCs. Future transplant experiments of the bone marrow may show detailed results. There remains a need to evaluate the persistent contribution to FPR-directed homing of BMSPCs (mobilizing speed, amount, etc.) toward the wound surface in the involvement in the provenance of marrow, then cells from the wound should be clearly picked and quantitatively analyzed.

## Conclusion

In summary, this is the new evidence that a coordinated change in glucocorticoid induced the coupling of FPR and CXCR4 guides the mobilization of BMSPCs after stress. These findings provide an important regulatory target for the selective mobilization of BMSPCs and have potential significance for tissue repair.

## Supplementary Information

**Additional file 1: Supplemental Fig. 1.** Relationship between the glucocorticoid levels with the genotype of CRH after acute running stress. ** *P* < 0.01 v.s. the Crh^+/+^ sham group, ^# #^ *P* < 0.01 v.s. the Crh^+/+^ stress group. *n*= 9-12 per group. Data are mean ± SD.

**Additional file 2: Supplemental Fig. 2.** Effects of changing glucocorticoid levels on the mobilization of bone marrow stem cells in vivo after acute running stress. a Relative circulating MSCs number. ** *P* < 0.01 v.s. the Crh^+/+^ sham group, ^# #^ P < 0.01 v.s. the Crh^+/+^ stress group. b Relative circulating EPCs number, c: Flow cytometry data of circulating MSCs, d: Flow cytometry data of circulating EPCs. ** *P* < 0.01 v.s. the Crh^+/+^ sham group, ^#^ *P* < 0.05 v.s. the Crh^+/+^ stress group. MSCs: mesenchymal stem cells; EPCs: endothelial progenitor cells. *n*= 9-15 per group. Data are mean ± SD.

**Additional file 3: Supplemental Fig. 3.** Prolonged glucocorticoid mini-infusion imitated the stress-induced increase in circulating MSCs and EPC in Crh ^+/+^ mice. a Relative circulating MSCs number. ** P < 0.01 v.s. the saline group. b Relative circulating EPCs number. ** P < 0.01 v.s. the saline group, c: Flow cytometry data of circulating MSCs, d: Flow cytometry data of circulating EPCs. MSCs: mesenchymal stem cells; EPCs: endothelial progenitor cells. n= 9-10 per group. Data are mean ± SD.

**Additional file 4: Supplemental Fig 4.** Prolonged glucocorticoid mini-infusion made up the impaired mobilization in circulating MSCs and EPCs in Crh ^-/-^ mice. a Relative circulating MSCs number. ** P < 0.01 v.s. the saline group. b Relative circulating EPCs number. * *P* < 0.05 v.s. the saline group, c: Flow cytometry data of circulating MSCs, d: Flow cytometry data of circulating EPCs. MSCs: mesenchymal stem cells; EPCs: endothelial progenitor cells. *n* = 6-7 per group. Data are mean ± SD.

**Additional file 5: Supplemental Fig 5.** Ex vivo glucocorticoid stimulation failed to change the number of innate circulating MSCs and EPC within the experimental time. a Relative circulating MSCs number. b Relative circulating EPCs number, c: Flow cytometry data of circulating MSCs, d: Flow cytometry data of circulating EPCs. MSCs: mesenchymal stem cells; EPCs: endothelial progenitor cells. *n*= 12 per group. Data are mean ± SD.

## Data Availability

The data that support the findings of this study are available from the corresponding author upon reasonable request.

## Abbreviations

BM: Bone marrow
BMSPCs: Bone marrow stem and progenitor cells
BMSCs: Bone marrow mesenchymal stem cells
Crh: Corticotropin-releasing hormone
CXCR4: Cys-X-Cys receptor 4
ELISA: Enzyme-linked immunosorbent assay
EPCs: Endothelial progenitor cells
FACS: Flow cytometric analyses
fMLP: N-formyl methionyl leucyl phenylalanine
FPR: N-formyl peptide receptor
GR: Glucocorticoid receptor
HPA: Hypothalamic-pituitary-adrenal
HPCs: Hematopoietic progenitor cells
SAM: Sympathetico-adrenomedullary
SDF-1α: Stromal cell-derived factor 1α
