# Supplementary figures and images for: Glucocorticoid guides mobilization of bone marrow stem/progenitor cells via FPR and CXCR4 coupling
Source: Stem Cell Res Ther. 2021 Jan 7;12:16. doi: 10.1186/s13287-020-02071-1 (PMC7791823; doi:10.1186/s13287-020-02071-1)

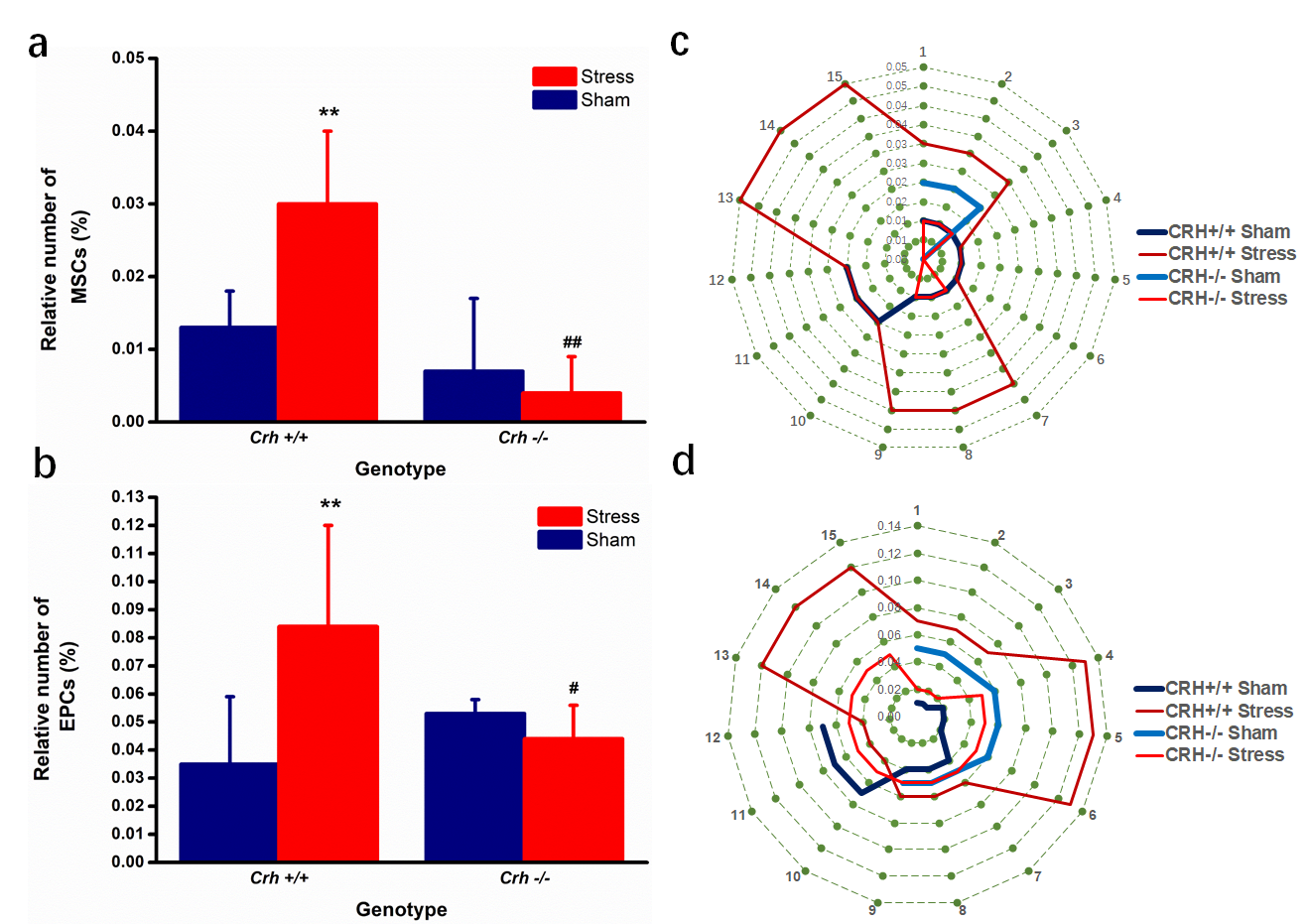

Supplement: Supplementary file 2 — Additional file 2: Supplemental Fig. 2. Effects of changing glucocorticoid levels on the mobilization of bone marrow stem cells in vivo after acute running stress. a Relative circulating MSCs number. ** P < 0.01 v.s. the Crh+/+ sham group, # # P < 0.01 v.s. the Crh+/+ stress group. b Relative circulating EPCs number, c: Flow cytometry data of circulating MSCs, d: Flow cytometry data of circulating EPCs. ** P < 0.01 v.s. the Crh+/+ sham group, # P < 0.05 v.s. the Crh+/+ stress group. MSCs: mesenchymal stem cells; EPCs: endothelial progenitor cells. n= 9-15 per group. Data are mean ± SD. [file 13287_2020_2071_MOESM2_ESM.tif]

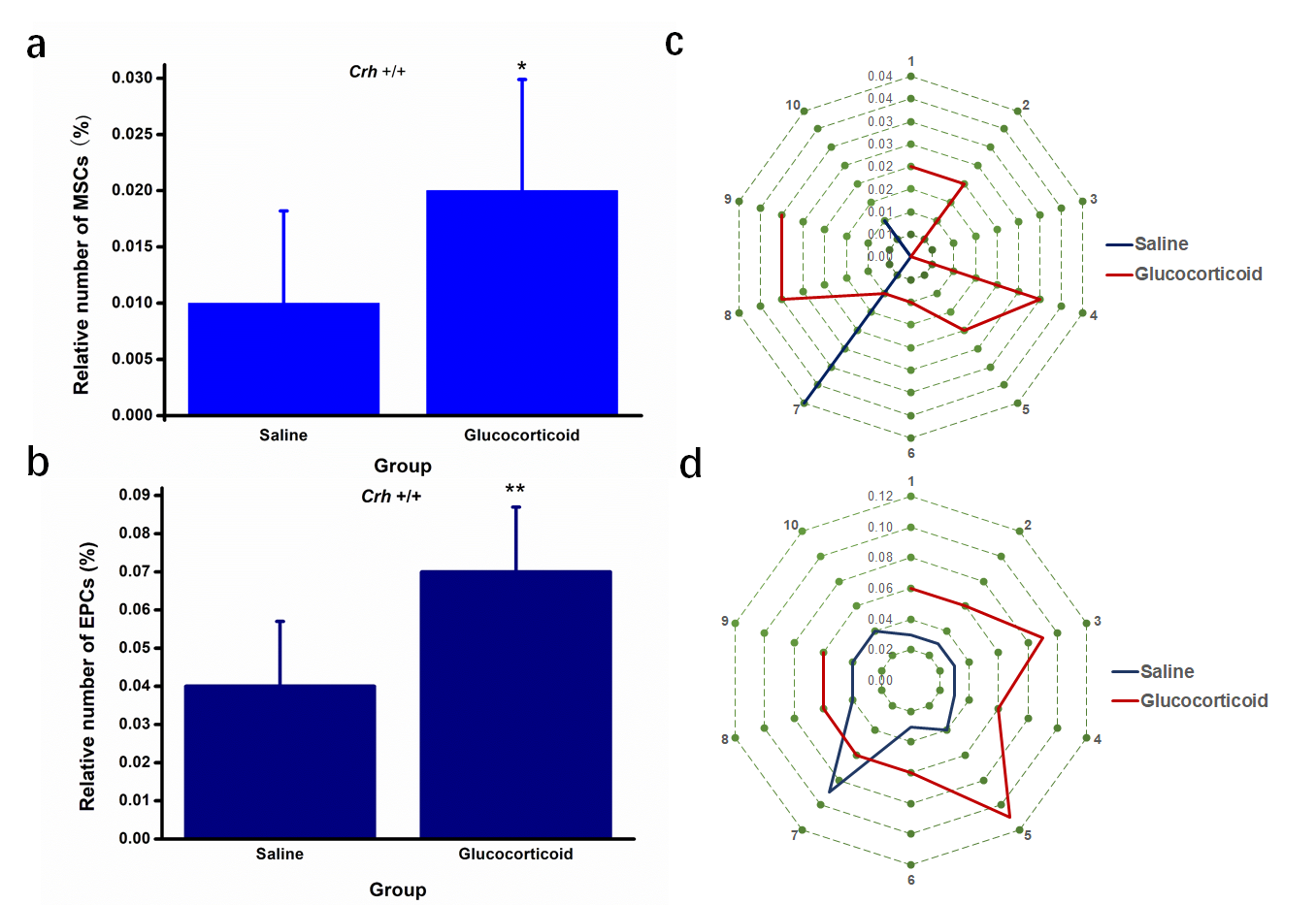

Supplement: Supplementary file 3 — Additional file 3: Supplemental Fig. 3. Prolonged glucocorticoid mini-infusion imitated the stress-induced increase in circulating MSCs and EPC in Crh +/+ mice. a Relative circulating MSCs number. ** P < 0.01 v.s. the saline group. b Relative circulating EPCs number. ** P < 0.01 v.s. the saline group, c: Flow cytometry data of circulating MSCs, d: Flow cytometry data of circulating EPCs. MSCs: mesenchymal stem cells; EPCs: endothelial progenitor cells. n= 9-10 per group. Data are mean ± SD. [file 13287_2020_2071_MOESM3_ESM.tif]

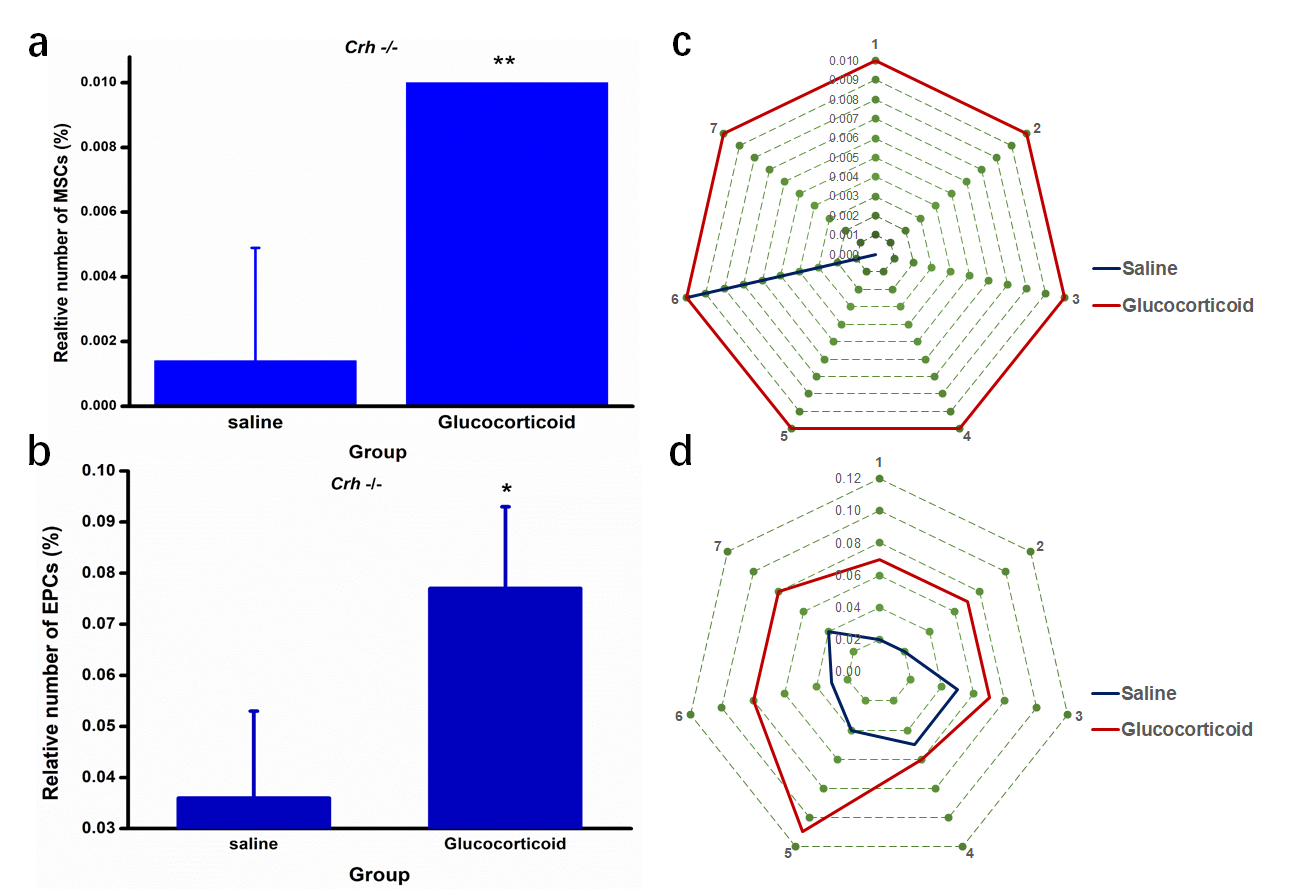

Supplement: Supplementary file 4 — Additional file 4: Supplemental Fig 4. Prolonged glucocorticoid mini-infusion made up the impaired mobilization in circulating MSCs and EPCs in Crh -/- mice. a Relative circulating MSCs number. ** P < 0.01 v.s. the saline group. b Relative circulating EPCs number. * P < 0.05 v.s. the saline group, c: Flow cytometry data of circulating MSCs, d: Flow cytometry data of circulating EPCs. MSCs: mesenchymal stem cells; EPCs: endothelial progenitor cells. n = 6-7 per group. Data are mean ± SD. [file 13287_2020_2071_MOESM4_ESM.tif]

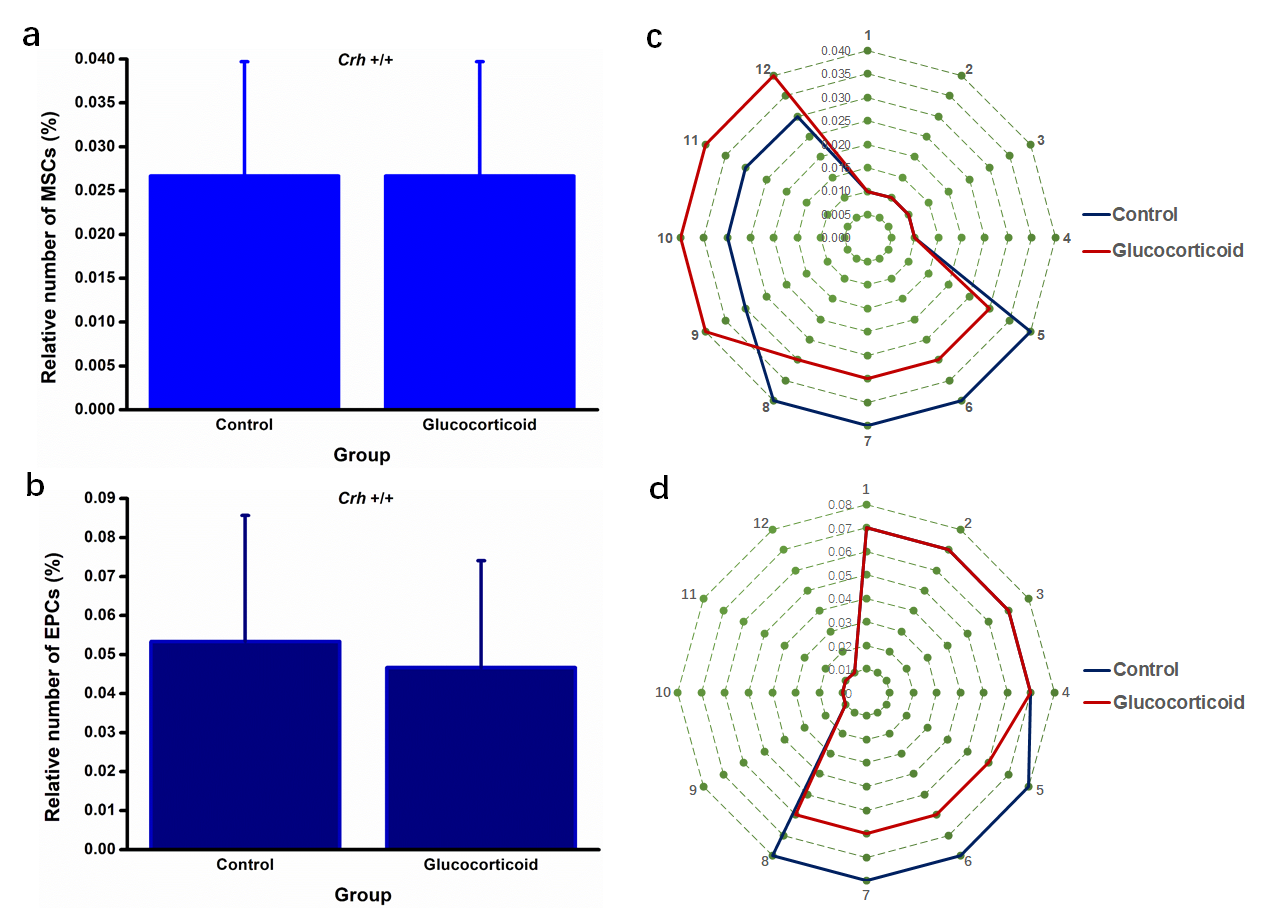

Supplement: Supplementary file 5 — Additional file 5: Supplemental Fig 5. Ex vivo glucocorticoid stimulation failed to change the number of innate circulating MSCs and EPC within the experimental time. a Relative circulating MSCs number. b Relative circulating EPCs number, c: Flow cytometry data of circulating MSCs, d: Flow cytometry data of circulating EPCs. MSCs: mesenchymal stem cells; EPCs: endothelial progenitor cells. n= 12 per group. Data are mean ± SD. [file 13287_2020_2071_MOESM5_ESM.tif]
